# Exercise training increases protein O‐GlcNAcylation in rat skeletal muscle

**DOI:** 10.14814/phy2.12896

**Published:** 2016-09-23

**Authors:** Kristin Halvorsen Hortemo, Per Kristian Lunde, Jan Haug Anonsen, Heidi Kvaløy, Morten Munkvik, Tommy Aune Rehn, Ivar Sjaastad, Ida Gjervold Lunde, Jan Magnus Aronsen, Ole M. Sejersted

**Affiliations:** ^1^ Institute for Experimental Medical ResearchOslo University Hospital and University of OsloOsloNorway; ^2^ Center for Heart Failure ResearchUniversity of OsloOsloNorway; ^3^ Department of BiosciencesUniversity of OsloOsloNorway; ^4^ Bjørknes CollegeOsloNorway

**Keywords:** Exercise, fatigue, heart failure, O‐GlcNAcylation, skeletal muscle

## Abstract

Protein O‐GlcNAcylation has emerged as an important intracellular signaling system with both physiological and pathophysiological functions, but the role of protein O‐GlcNAcylation in skeletal muscle remains elusive. In this study, we tested the hypothesis that protein O‐GlcNAcylation is a dynamic signaling system in skeletal muscle in exercise and disease. Immunoblotting showed different protein O‐GlcNAcylation pattern in the prototypical slow twitch soleus muscle compared to fast twitch EDL from rats, with greater O‐GlcNAcylation level in soleus associated with higher expression of the modulating enzymes O‐GlcNAc transferase (OGT), O‐GlcNAcase (OGA), and glutamine fructose‐6‐phosphate amidotransferase isoforms 1 and 2 (GFAT1, GFAT2). Six weeks of exercise training by treadmill running, but not an acute exercise bout, increased protein O‐GlcNAcylation in rat soleus and EDL. There was a striking increase in O‐GlcNAcylation of cytoplasmic proteins ~50 kDa in size that judged from mass spectrometry analysis could represent O‐GlcNAcylation of one or more key metabolic enzymes. This suggests that cytoplasmic O‐GlcNAc signaling is part of the training response. In contrast to exercise training, postinfarction heart failure (HF) in rats and humans did not affect skeletal muscle O‐GlcNAcylation level, indicating that aberrant O‐GlcNAcylation cannot explain the skeletal muscle dysfunction in HF. Human skeletal muscle displayed extensive protein O‐GlcNAcylation that by large mirrored the fiber‐type‐related O‐GlcNAcylation pattern in rats, suggesting O‐GlcNAcylation as an important signaling system also in human skeletal muscle.

## Introduction

Modification of intracellular proteins by N‐acetylglucosamine (GlcNAc), known as O‐GlcNAcylation, is a reversible and dynamic posttranslational modification (PTM) that was first described in 1984 (Torres and Hart 1984). In recent years, protein O‐GlcNAcylation has emerged as an important signaling system with both physiological and pathological functions (Hart et al. 2007, 2011; Hardiville and Hart 2014; Marsh et al. 2014; Bond and Hanover 2015). Several skeletal muscle proteins have been identified as O‐GlcNAc modified (Cieniewski‐Bernard et al. 2004; Hedou et al. 2007), and we propose that protein O‐GlcNAcylation in skeletal muscle may be related to fiber type, exercise and disease.

The two enzymes O‐GlcNAc transferase (OGT) and O‐GlcNAcase (OGA) add and remove the GlcNAc via an O‐linkage to serine/threonine residues of cytoplasmic, nuclear, and mitochondrial proteins. O‐GlcNAcylation is in many ways analogous to O‐phosphorylation by kinases and phosphatases, and there is evidence to suggest a fine interplay between protein O‐GlcNAcylation and O‐phosphorylation (Butkinaree et al. 1800; Hart et al. 2011). However, the modulation of O‐GlcNAcylation by only two conserved enzymes, OGT and OGA (Hart et al. 2007), contrasts the many hundred kinases and phosphatases regulating phosphorylation. The substrate for O‐GlcNAcylation is uridine diphosphate N‐acetylglucosamine (UDP‐GlcNAc), which is the end product of the hexosamine‐biosynthetic pathway (HBP). A small fraction of glucose entering the cell is diverted into the HBP as an alternative route to glycolysis (Marshall et al. 1991), controlled by the rate‐limiting enzyme glutamine fructose‐6‐phosphate amidotransferase (GFAT).

O‐GlcNAc is a cellular sensor for metabolic status, interacting with glucose, amino acid, fatty acid, and nucleotide metabolic pathways (Hardiville and Hart 2014). During exercise, glucose uptake in skeletal muscle is increased. This increased glucose uptake is likely to increase the flux through the HBP, since glucose is the paramount regulator of this pathway (Marshall et al. 1991). Accordingly, it was reported that the end product of the HBP (i.e., UDP‐GlcNAc) increased in rat skeletal muscle after acute exercise (Nelson et al. 1997). However, the effects of exercise on protein O‐GlcNAcylation in skeletal muscle are not well understood (Myslicki et al. 2014). Only a few studies have investigated skeletal muscle O‐GlcNAc signaling in exercise (Nelson et al. 1997; Toivonen et al. 2013; Hortemo et al. 2015; Peternelj et al. 2015), and the effects of long‐term exercise on protein O‐GlcNAcylation have not been explored. The extent of protein O‐GlcNAcylation in the predominantly oxidative slow twitch muscles (type I fibers) versus the more glycolytic fast twitch muscles (type II fibers) is also poorly mapped. In skeletal muscle myofilaments, the regulatory protein myosin light chain 2 (MLC2) can be dynamically modified by both phosphorylation and O‐GlcNAcylation (Hortemo et al. 2013, 2015; Stevens et al. 2013; Cieniewski‐Bernard et al. 2014) in response to the activity level of the muscle, but the extent of O‐GlcNAc signaling on other myofilament proteins in response to exercise remains to explore.

In cardiac muscle, Lunde et al. (2012) previously reported increased O‐GlcNAc signaling in cardiac pressure overload and heart failure (HF), and several other studies suggest that a sustained increase in protein O‐GlcNAcylation is detrimental to the heart (reviewed by Marsh et al. (2014). Furthermore, a hallmark of HF is reduced exercise tolerance, which in part is attributed to skeletal muscle dysfunction (Zizola and Schulze 2013). Previous studies from our research group have reported skeletal muscle dysfunction in postinfarction HF, both in rats (Lunde et al. 2001, 2002, 2006) and humans (Munkvik et al. 2010). Whether altered skeletal muscle O‐GlcNAcylation contributes to this dysfunction, parallel to the aberrant O‐GlcNAcylation in the heart, is not known.

In this study, we aimed to map the overall protein O‐GlcNAcylation pattern in resting slow and fast twitch muscles, and to explore the effects of acute and long‐term exercise training on skeletal muscle protein O‐GlcNAcylation. Finally, we investigated if aberrant protein O‐GlcNAcylation is part of the skeletal muscle dysfunction in postinfarction HF.

## Methods

### Ethical approval

All animal experiments were performed in accordance with the Norwegian Animal Welfare Act. Protocols were reviewed and approved by the Norwegian Animal Research Authority (ID 1543 and 3301) and conformed to the NIH Guide for the Care and Use of Laboratory Animals. Male Wistar and Sprague Dawley rats (Taconic, Skensved, Denmark) were housed in a controlled environment (temperature 22 ± 2°C, humidity 55 ± 5%, 12/12‐h daylight/night cycle) for 1 week before study inclusion. Rats were fed standard rat chow (B & K Universal, Oslo, Norway) and water ad libitum. The human study complied with the Declaration of Helsinki and was approved by the Regional Committee for Medical and Health Research Ethics. Written informed consent was obtained from all participants.

### Treadmill running – 6 weeks of exercise training

Male Sprague Dawley rats ~300 g (*n* = 16) were randomly assigned to perform a 6 weeks interval training program (run) on the treadmill (Columbus Instruments, Columbus, OH) or to remain sedate (control). The training program was modified from Wisloff et al. (2001). Sprague Dawley rats were used in this set of experiments because this was the required strain for a parallel study on cardiac muscle (unpubl. results). Six days of familiarization to treadmill running was performed with a running velocity of 6 m min^−1^ for 30, 45, 60, 75, 90, and 120 min, respectively, and 1 day of rest. Interval training was then performed 6 days a week at 25 degrees inclination on the treadmill; 10 min warm‐up (10 m min^−1^) followed by 12 × 8 min intervals separated by 2 min resting periods (6 m min^−1^). The running speed during intervals was 15 m min^−1^ the first week, then increasing with 2 m min^−1^ each week, reaching 25 m min^−1^ after 6 weeks. The exercised rats had lower body weight compared to sedate controls after 6 weeks (run 345 ± 8, *n* = 8 vs. control 438 ± 11 g, *n* = 8, *P* < 0.05). Development of cardiac hypertrophy in the exercised rats (heart weight 1.30 ± 0.03 vs. control 1.16 ± 0.01 g; heart weight/body weight 3.80 ± 0.09 vs. control 2.64 ± 0.03, both *P* < 0.05) showed cardiovascular adaption to exercise training. Resting heart rate was not different between groups (run 346 ± 14 vs. control 382 ± 11). Rats were given 0.1–0.2 g chocolate (Kvikk Lunsj, Freia, Oslo, Norway) after each training session. Rats not able to fulfill the exercise protocol were excluded from the study. After the last training session (after 6 weeks), rats were allowed 24 h rest before muscles were harvested. Rats were anesthetized in a chamber and subsequently mask ventilated by 3% isoflurane in O_2_, and within 3 min after onset of anesthesia the soleus and EDL muscles were dissected free and snap‐frozen in liquid nitrogen before the animals were sacrificed by cardiac excision while still anesthetized.

### Treadmill running – acute exercise

Male Wistar rats ~300 g (*n* = 18) were familiarized to running on the treadmill for 15 min the last 2 days prior to the experiment (5 min at 8 m min^−1^, 10 min at 12 m min^−1^). This rat cohort was also included in a previous work from our laboratory (Hortemo et al. 2015). At the day of the experiment, rats were randomly assigned to three different groups; the acute exercise group performed an acute exercise bout to fatigue, the recovery group performed an acute exercise bout to fatigue and was subsequently allowed 24 h rest, and the control group remained sedate. Exercise was performed at 12.5 degrees inclination with incremental running speed, starting at 8 m min^−1^ and increasing every second min toward maximum running speed of the individual rat. The exercise protocol was continued until exhaustion, defined as when the rat was unable to continue running at the maximum speed. Animals in the running group reached a maximum running speed of 20 ± 1 m min^−1^, and the time to exhaustion was 26 ± 1 min. Rats in the acute exercise group were at the end of exercise immediately anesthetized in a chamber with 4% isoflurane (Forene^®^, Abbvie, Fornebu, Norway) in air and sacrificed by neck dislocation (cardiac excision was not performed in this group, as no hearts were harvested). Within 1 min after termination of the exercise protocol, soleus and extensor digitorum longus (EDL) muscles were harvested and snap‐frozen in liquid nitrogen and stored at −80°C until analysis. Rats in the recovery group were at the end of exercise allowed 24 h rest before muscles were harvested as described above.

### Postinfarction heart failure in rats

Myocardial infarction was induced by left coronary artery ligation. Male Wistar rats ~300 g (*n* = 7) were sedated with a mixture of 4% isoflurane, 64% N_2_O, and 32% O_2_ in an anesthesia chamber, before ventilated through endotracheal intubation coupled to a Zoovent ventilator (Triumph Technical Services, Milton Keynes, UK). Lateral thoracotomy was performed through the fourth intercostal space, before the pericardium was opened. The left anterior descending coronary was identified and ligated with a 5.0‐silk suture. Large anterolateral infarction was visually confirmed, before closing the chest with muscle and skin sutures. Extubation was performed after recovery of spontaneous ventilation, and rats were kept in a heated incubator for 30–60 min after the procedure. Sham‐operated rats (*n* = 7) underwent the same procedure, except the ligation of the coronary artery, and served as controls. Buprenorphine was given as postoperative analgesia.

Echocardiographic examinations were performed 6 weeks after induction of myocardial infarction using the Vevo2100 system (VisualSonics Inc, Toronto, ON, Canada) in light anesthesia (2% isoflurane in O_2_) delivered by mask ventilation, and congestive HF was confirmed by the presence of a large anterolateral myocardial infarction in the left ventricle and increased atrial diameter (HF 6.5 ± 0.3 vs. sham 3.6 ± 0.2 mm, *P* < 0.05) (Sjaastad et al. 2000). Two days later, the rats were deeply anesthetized with 3–4% isoflurane in O_2_, and the soleus and EDL muscles were harvested and snap‐frozen in liquid nitrogen. Subsequently, congestive HF was confirmed by increased lung weight (HF 4.13 ± 0.43 vs. sham 1.52 ± 0.24 g, *P* < 0.05)) and heart weight (HF 3.05 ± 0.25 vs. sham 1.62 ± 0.06 g, *P* < 0.05), while body weight did not differ between groups (HF 418 ± 4 vs. sham 398 ± 21 g).

### Heart failure patients and healthy subjects

Skeletal muscle biopsies were obtained from vastus lateralis in male patients with heart failure (HF) and in healthy control subjects (HS) (*n* = 6 + 6). The human HF patients represented the same subtype of HF as in the rat model, that is, postinfarction HF with reduced ejection fraction (HFrEF). Percutaneous needle biopsies were performed under sterile conditions with local anesthesia (Xylocaine–Adrenaline, 10 mg mL^−1^ + 5 *μ*g mL^−1^, AstraZeneca, Oslo, Norway) using a 6‐mm Pelomi needle (Albertslund, Denmark) with manual suction, and the biopsies were frozen in isopentane on dry ice and stored at −80°C. Age and weight was not different between HF patients and HS (HF 67 ± 2 vs. HS 70 ± 3 years old; HF 82 ± 7 vs. HS 87 ± 4 kg), while HF patients had reduced ejection fraction (HF 28 ± 4 vs. HS 68 ± 1%, *P* < 0.001) as well as lower maximal oxygen uptake (HF 19 ± 3 vs. HS 28 ± 2 mL kg^−1^ min^−1^, *P* < 0.05). HF patients had reduced skeletal muscle peak torque (HF 136 ± 10 vs. HS 168 ± 12 Nm, *P* < 0.05) and peak power (HF 27 ± 3 vs. HS 35 ± 3 W, *P* < 0.05) of vastus lateralis compared to HS. The human subjects in this study were a subset of participants from a previous study from our laboratory, and details about the methodology, testing regime, and inclusion/exclusion criteria have been described (Munkvik et al. 2010).

### Protein extraction

**Figure 1:**
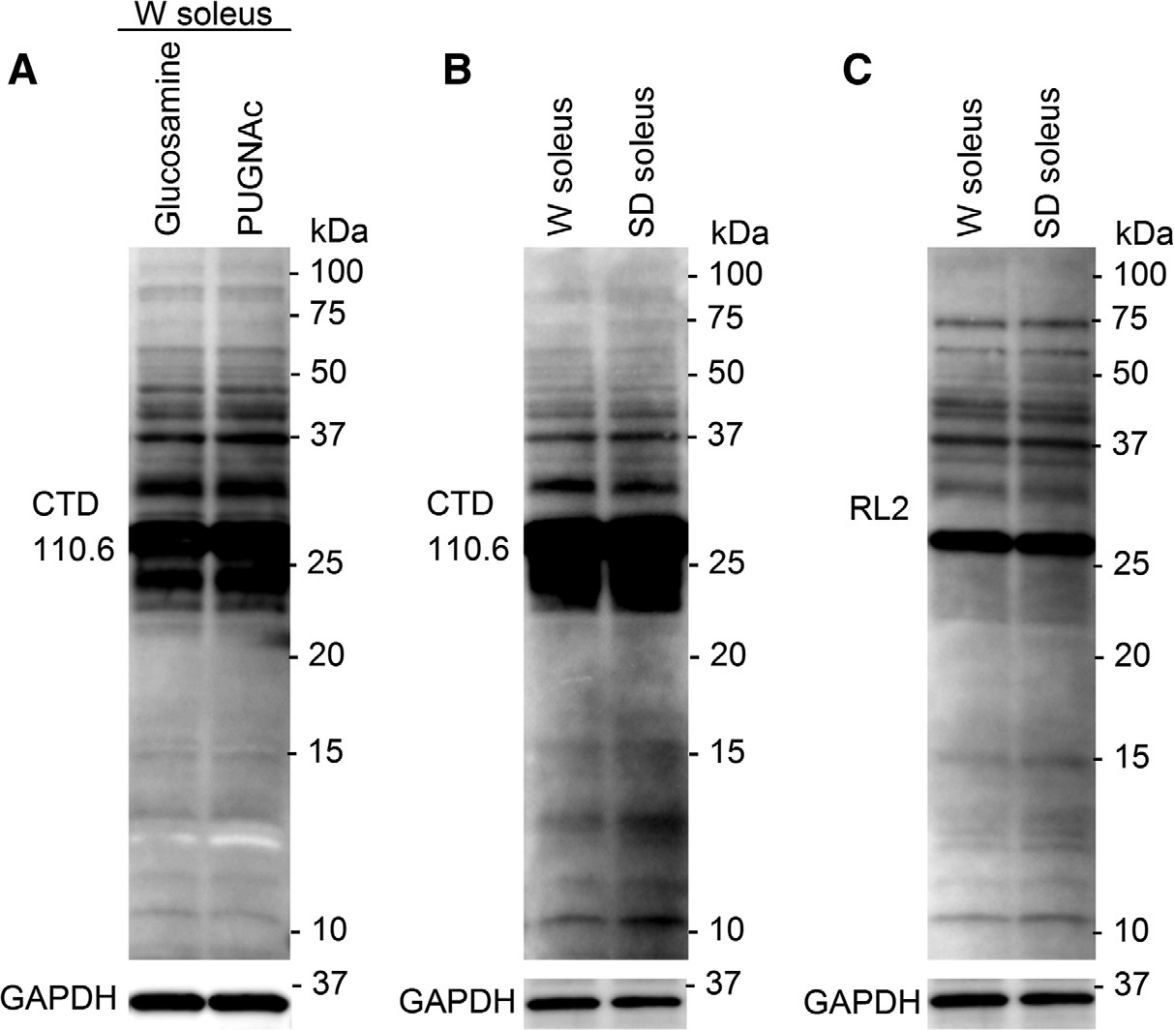
Control for potential effects of different OGA inhibitors, rat strains, and O‐GlcNAc antibodies on protein O‐GlcNAcylation. Muscle cell lysates of soleus from Wistar (W) containing glucosamine was compared to lysates containing the OGA inhibitor PUGNAc (A), revealing identical O‐GlcNAcylation patterns by the two methods. Furthermore, muscle cell lysates of soleus from both Wistar and Sprague Dawley (SD) rats were analyzed for O‐GlcNAcylation pattern using the CTD110.6 antibody (B), showing no differences between the strains. Finally, parallel analysis of the same samples as in B with the RL2 antibody (C) showed a slightly different O‐GlcNAcylation pattern compared to B, as expected from the literature. However, neither the RL2 antibody revealed any differences in O‐GlcNAcylation pattern between the rat strains (*n* = 3).

Total protein lysates were made by pulverizing muscles in a mortar with liquid nitrogen and subsequently homogenizing with a Polytron^®^ 1200 (Kinematica, Luzern, Switzerland) in ice‐cold lysis buffer (20 mmol L^−1^ Hepes pH 7.5, 150 mmol L^−1^ NaCl, 1 mmol L^−1^ EDTA, 0.5% Triton X‐100) with protease inhibitors (Complete EDTA‐free tablets, Roche Diagnostics, Oslo, Norway), phosphatase inhibitors (PhosSTOP, Roche Diagnostics) and 40 mmol L^−1^ glucosamine (Sigma‐Aldrich, Oslo, Norway). Addition of glucosamine to provide excess substrate for OGA previously resulted in O‐GlcNAc immunoblots of very good quality (Lunde et al. 2012). In a test run, we compared the addition of glucosamine to the addition of the OGA‐inhibitor PUGNAc (40 *μ*mol L^−1^) (Tocris) (Fig. 1A), revealing similar O‐GlcNAcylation patterns by the two approaches. The homogenates were stored on ice for 30 min and centrifuged at 20,000 *g* for 30 min at 4°C. Supernatants were stored at −80°C until analysis.

Myofibrillar protein extracts were made by pulverizing muscles in a mortar with liquid nitrogen (Hortemo et al. 2015). Ice‐cold 6.35 mmol L^−1^ EDTA solution with protease inhibitors, phosphatase inhibitors, and 40 mmol L^−1^ glucosamine was added and the samples homogenized with Polytron^®^ 1200, stored on ice for 30 min, and centrifuged at 18,000 *g* for 10 min at 4°C. Pellets were washed with 50 mmol L^−1^ KCl containing protease inhibitors, phosphatase inhibitors, and glucosamine, and centrifuged for another 10 min. The final pellets were resuspended in 50 mmol L^−1^ KCl containing protease inhibitors, phosphatase inhibitors, and glucosamine, and stored at −80°C until analysis. Successful fractionation of myofilaments proteins was described in a previous study (Hortemo et al. 2015).

### Immunoblotting

Protein concentrations were determined using the Micro BCA Protein Assay Kit (Pierce/Thermo Scientific, Oslo, Norway) and 20–90 *μ*g of protein was loaded onto 1.0 mm 4–15% or 15% Tris‐HCl gels (Criterion, BIO‐RAD, Oslo, Norway). In a pilot study, we found O‐GlcNAc modifications of skeletal muscle proteins predominantly in the range 20–100 kDa, and we therefore focused our study on proteins in this size range. SDS‐PAGE and western blotting was performed essentially as described in the Criterion BIORAD protocol, using PVDF Hybond membranes (Amersham/GE Healthcare, Oslo, Norway). Blots were blocked in either 5% nonfat dry milk or 5% BSA for 1 h at room temperature, and incubated with primary and secondary antibodies overnight at 4°C and 1 h at room temperature, respectively.

For the analysis of protein O‐GlcNAcylation level, we used the O‐GlcNAc antibody CTD110.6 (MMS‐248R, Covance, Oslo, Norway) that was reported to recognize the widest range of O‐GlcNAc‐modified proteins and was the most thoroughly characterized (Zachara 2009). However, it is known that different O‐GlcNAc antibodies do not recognize exactly the same O‐GlcNAc modifications (Zachara 2009), and we compared the antibody CTD110.6 with the antibody RL2 (MA1‐072, Thermo Scientific) (Fig. 1B and C). As expected, many protein bands were detected by both antibodies, but there were also differences in the O‐GlcNAcylation patterns. A previous publication from our research group (Lunde et al. 2012) successfully used the CTD110.6 antibody to obtain high‐quality O‐GlcNAcylation blots in cardiac muscle. Furthermore, Reeves et al. (2014) recently investigated the potential for CTD110.6 to cross‐react with N‐GlcNAc2, and concluded that this nonspecificity of CTD110.6 was only present in conditions of glucose deprivation that appeared to be nonphysiological. No other type of cell stress induced N‐GlcNAc2‐modifications and, based on their results, the CTD110.6 could be considered an O‐GlcNAc‐specific antibody in the physiological settings of the present study.

The other primary antibodies used were anti‐OGT (O6264, Sigma‐Aldrich), anti‐OGA (SAB4200267, Sigma‐Aldrich), anti‐GFAT1 (ab125069, Abcam, Cambridge, UK), anti‐GFAT2 (sc‐134710; Santa Cruz Biotechnology (SCB), Heidenberg, Germany), anti‐glyceraldehyde 3‐phosphate dehydrogenase (GAPDH) (sc‐20357, SCB), anti‐myosin light chain 2 (MLC2) (F109.3E1, BioCytex, Oslo, Norway), anti‐*α*‐tubulin (sc‐5286, SCB), anti‐citrate synthase (CS) (ab96600, abcam), anti‐phospho‐serine (61‐8100, Invitrogen), and anti‐phospho‐threonine (9381, Cell Signaling Technology, Leiden, The Netherlands). Blots were incubated with appropriate anti‐HRP‐conjugated secondary antibodies (Southern Biotechnology, Birmingham, AL), developed using the ECL Plus Western Blotting Detection System (Amersham/GE Healthcare) and visualized in a Las‐4000 mini (Fujifilm, Stockholm, Sweden). Blots were reprobed after stripping using the Restore Western Blot Stripping Buffer (21059, Thermo Scientific). The efficiency of the stripping protocol was demonstrated in a previous study (Hortemo et al. 2015).

**Figure 2:**
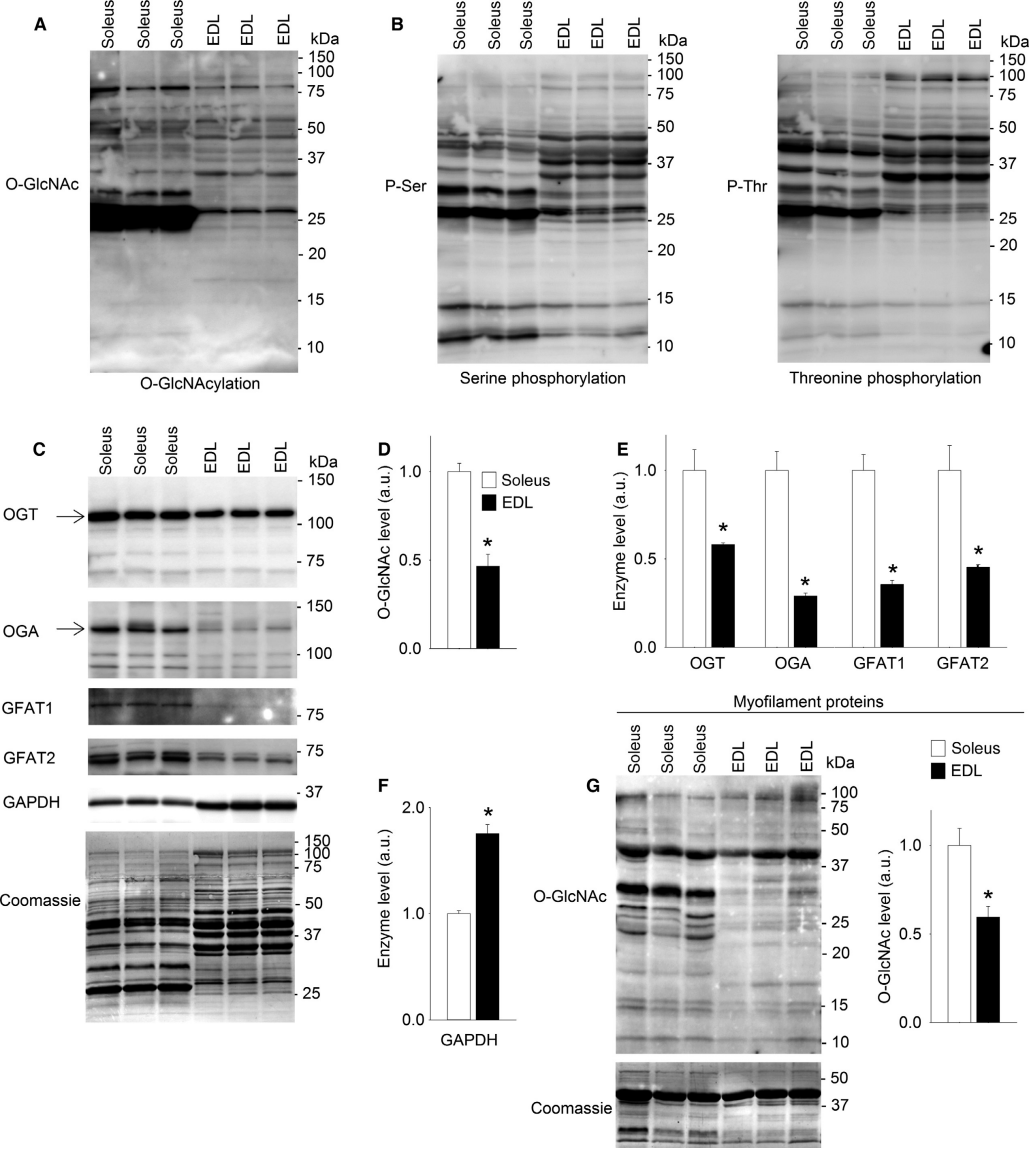
Higher levels of protein O‐GlcNAcylation in slow twitch than fast twitch skeletal muscle. Total protein lysates analyzed by immunoblotting for O‐GlcNAcylation (A), protein phosphorylation at serine and threonine residues (B), and O‐GlcNAc transferase (OGT), O‐GlcNAcase (OGA), glutamine fructose‐6‐phosphate amidotransferase isoforms 1 (GFAT1), and glutamine fructose‐6‐phosphate amidotransferase isoforms 2 (GFAT2), and GAPDH enzymes (C) from resting slow twitch soleus and fast twitch EDL (Wistar rats). Average data (D, E, and F) shown as mean ± SEM, with staining intensities in EDL (black bars) calculated relative to soleus (white bars). O‐GlcNAcylation of myofilament proteins (G) analyzed by immunoblotting, and shown as average O‐GlcNAcylation level in EDL calculated relative to soleus. Equal loading was confirmed by Coomassie staining. Arrows indicate the bands included in the calculation of OGT and OGA (*n* = 3. *P <* *0.05 vs. soleus).

Quantification of protein band intensity and processing of immunoblots were performed using ImageQuant (GE Healthcare) and Adobe Photoshop CS5. Quantification of overall O‐GlcNAcylation level was performed by densitometry of entire lanes. However, in the analysis of soleus total homogenate the intense immunoreactive band at ~25 kDa was excluded since this band dominated the overall densitometry signal (and the band was not regulated by exercise or by heart failure, controlled by quantification at shorter exposure time). The signal strength of the entire lane (or of specific bands) was normalized to the loading control of each sample. GAPDH level was not altered after exercise training compared to control, and was used as loading control for total protein lysates. Correspondingly, tubulin was used for myofilament proteins. Coomassie staining was used to confirm equal loading when comparing different muscle types, because several of the standard proteins used for loading control were differently expressed between the muscle types (e.g., GAPDH, Fig. 2C).

Fluorescent gel stains ProQ Diamond and SYPRO Ruby (Molecular Probes, Invitrogen, Oslo, Norway) were used for sequential analysis of protein phosphorylation and total proteins levels, respectively, for comparison with the O‐GlcNAc level especially at ~50 kDa. Bands were detected by a Typhoon laser scanner (Typhoon 9410, GE Healthcare, Oslo, Norway), and staining intensity was quantified by ImageQuant.

In all comparisons between samples, we used same strain controls (e.g., muscles from exercised Wistar rats were compared to muscles from control Wistar rats). However, a comparison of the O‐GlcNAc pattern in skeletal muscles from Wistar versus Sprague Dawley rats (Fig. 1B and C) revealed identical O‐GlcNAcylation pattern between the strains.

### Subcellular protein fractioning

Tissue from soleus muscle (25 mg) was homogenized with Polytron^®^ 1200 and sequentially isolated into cytoplasmic, nuclear and membrane, and cytoskeletal proteins by the use of Compartmental Protein Extraction Kit (cat. no. 2145, Millipore/Merck Life Sciences, Oslo, Norway). Anti‐GAPDH was used as a marker for cytoplasmic proteins, anti‐sarco/endoplasmic reticulum Ca^2+^‐ATPase (SERCA2) (MA3‐919, Thermo Scientific) for membrane proteins, anti‐succinate dehydrogenase (SDH) (sc‐27992, SCB) for mitochondrial proteins, anti‐MLC2 for myofilament proteins, and anti‐histone H3 (H3) (sc‐10809, SCB) for nuclear proteins.

### In‐gel digest

Enriched cytoplasmic proteins (130 *μ*g) from exercised (6 weeks exercise training) and control soleus muscles were loaded in separate lanes onto a 10% SDS‐PAGE gel. The gel was Coomassie stained and subsequently aligned to an identically loaded immunoblot with anti‐O‐GlcNAc antibody. Correct alignment was confirmed by two researchers experienced with gel analysis, establishing the gel area corresponding to the anti‐O‐GlcNAc reactive band at ~50 kDa, and the gel pieces of interest were identified and cut from both the exercise and control samples. Proteinase digestion was carried out with 25 *μ*L trypsin (approximately 16 ng *μ*L^−1^, Sigma) in 50 mmol L^−1^ Na_2_HCO_3_ pH 7.8 incubated ON at 37°C. Destaining of Coomassie‐stained gel slices, reduction, alkylation, and extraction of protease‐generated peptides were performed as described previously (Anonsen et al. 2012). The extracted peptides were dried in a speed‐vac prior to nanoflow liquid chromatography coupled to tandem mass spectrometry (nano‐LC‐MS2) analysis.

### Reverse phase LC‐MS2 analysis

Reverse phase nano‐LC‐MS2 of proteolytically derived peptides was performed using a system consisting of a Dionex UltiMate 3000 UHPLC binary pumps (nano and capillary) with auto sampler, column heater, and integrated switching valve. The LC system was coupled via a nano electrospray ion source (nESI) to a QExactive mass spectrometer (Thermo Fisher Scientific, Bremen, Germany). For the analyses, 1 *μ*L of peptide solution was injected into the 5 × 0.3 mm extraction column filled with pepmap C18 of 0.3 mm i.d. and 5 *μ*m particle size (*μ*‐precolumn). Samples were washed with mobile phase (97% H_2_O/0.25% formic acid/3% acetonitrile). The flow rate was 15 *μ*L min^−1^ provided by the capillary pump. After 5 min, the integrated switching valve was activated and peptides were eluted in the back‐flush mode from the extraction column onto a 50 × 0.075 mm C18, 3 *μ*m resin column (Acclaim pepmap 100). The mobile phase consisted of acetonitrile and MS grade water, both containing 0.25% formic acid. Chromatographic separation was achieved using a binary gradient from 5% to 55% of acetonitrile in water in 60 min for peptide samples generated from in‐gel digestion. The flow rate was 0.3 *μ*L min^−1^ provided by the nanoflow pump. Nanospray ionization was achieved by applying 2.0 kV between a 8‐*μ*m diameter emitter (PicoTip Emitter, New Objective, Woburn, MA) and the capillary entrance of the Orbitrap. Mass spectra were acquired in the positive ion mode applying a data‐dependent automatic switch between survey scan and MS2 acquisition on the Thermo Scientific QExactive mass spectrometer operated by Xcalibur 3.0. Peptide samples were analyzed with higher energy collision dissociation (HCD) fragmentation method, acquiring one Orbitrap survey scan in the mass range of m/z 400–2000 followed by MS2 of the 12 most intense ions. The target value in the Orbitrap was 1,000,000 for survey scan at a resolution of 70,000 at m/z 400 using lock masses for recalibration to improve the mass accuracy of precursor ions. Fragmentation was performed by HCD with a target value of 5000 ions at a resolution of 17,500 at m/z 400 and a fixed first mass set at 150.0 m/z. Ion selection threshold was 500 counts. Selected sequenced ions were dynamically excluded for 60 s.

### Peptide and protein identification

All generated.raw files were manually searched for GlcNAc reporter ions at *m/z* 204.087 and *m/z* 186.076 employing Thermo Xcalibur software, Qual browser 3.0 (Thermo Fisher Scientific). Peptide and protein identification were performed using MaxQuant (Ver. 1.4.1.2, Max Planck Institute of Biochemistry, Munich, Germany) searched against an in‐house *Rattus norvegicus* protein FASTA sequence database generated from the National Center for Biotechnology (NCBI). Precursor ion mass tolerance were set to 20 ppm for first search and a product ion mass tolerance of 0.5 Da was used with a maximum of two missed trypsin cleavage sites. Cysteine carbamidomethylation was set as fixed modification and protein N‐terminal acetylation as well as oxidation of methionine residues as dynamic modifications. O‐linked (serine, threonine) GlcNAc modification of 203.079 Da was set as a dynamic modification. Peptides were identified using a target‐decoy approach with a peptide false discovery rate (FDR) of 1%.

The list generated from the MaxQuant search of the nano‐LC‐MS2 analysis was filtered to remove contaminants, proteins identified from single peptides or only in a single sample. Proteins in the molecular weight range 40–65 kDa identified by ≥2 peptides in samples from both exercised and control muscles in two independent MS analyses were considered potential positive identifications. Proteins 40–65 kDa in size were included to avoid erroneous rejection of the protein of interest, since theoretical and empirical (i.e., as visualized on the western blot) molecular weights can be divergent. Proteins categorized as nonskeletal muscle and/or noncytoplasmic proteins using the UniProt database (http://www.uniprot.org/) were excluded. The remaining proteins were functionally classified based on their annotation in Kyoto Encyclopedia of Genes and Genomes (KEGG) BlastKOALA (http://www.kegg.jp/blastkoala/) (Kanehisa et al. 2015). Previously reported O‐GlcNAc modifications of the identified proteins were searched for in dbOGAP v1.0 (http://cbsb.lombardi.georgetown.edu/hulab/OGAP.html), and prediction of O‐GlcNAc sites were performed by OGlcNAcScan (http://cbsb.lombardi.georgetown.edu/hulab/OGAP.html) (Wang et al. 2011) and YinOYang 1.2 (http://www.cbs.dtu.dk/services/YinOYang) (Gupta and Brunak 2002).

### Statistics

Data are expressed as means ± SEM relative to control, if not otherwise specified. For all tests, *P *<* *0.05 was considered significant. Differences between two groups were tested using two‐tailed Student's unpaired *t*‐test. The statistical analyses and the preparation of graphs were performed by means of SigmaPlot (Systat Software Inc, version 12.5, Erkrath, Germany) or Microsoft Excel 2010 (Microsoft, Oslo, Norway).

## Results

### Higher level of protein O‐GlcNAcylation in slow twitch than fast twitch skeletal muscle

Protein O‐GlcNAcylation in resting muscle was extensive in both slow twitch soleus and fast twitch EDL as evident by immunoblotting (Fig. 2A). The most abundant O‐GlcNAcylation was observed on proteins in the range 25–75 kDa. The pattern of protein O‐GlcNAc modification was different in the two muscle types, with overall O‐GlcNAcylation more than twofold higher in soleus compared to EDL (Fig. 2A and D). Since O‐GlcNAc modifications appear on serine and threonine residues of proteins, we made parallel immunoblots showing the pattern of serine and threonine phosphorylation (Fig. 2B). Overall, there was relatively little phosphorylation of proteins >50 kDa in size, while there was abundant phosphorylation of proteins in the range 25–50 kDa.

In accordance with the extensive O‐GlcNAcylation observed in soleus, the level of nucleocytoplasmic OGT (OGT, 110 kDa) was almost twofold higher in soleus compared to EDL (Fig. 2C and E). The levels of nucleocytoplasmic OGA (OGA, 130 kDa) and both isoforms of the rate‐limiting enzyme in the HBP, GFAT1 (79 kDa), and GFAT2 (~72 kDa, evident as a double band) were also higher in soleus compared to EDL. The glycolytic phenotype of EDL compared to soleus was verified by higher level of GAPDH (Fig. 2F). The calculation of OGT and OGA enzyme levels was based on the strongly expressed nucleocytoplasmic isoforms (Fig. 2C, arrows). However, in both soleus and EDL, there were additional, weak OGT‐immunoreactive bands (Fig. 2C) that could correspond to short (74 kDa) and mitochondrial (103 kDa) OGT (Butkinaree et al. 1800; Hanover et al. 2003). A weak OGA‐immunoreactive band at ~80 kDa could represent short OGA (75 kDa) (Butkinaree et al. 1800). We subsequently analyzed proteins in the myofilament fraction to investigate the subcellular distribution of protein O‐GlcNAcylation. There was widespread O‐GlcNAcylation also on myofilament proteins (Fig. 2G), with the most prominent O‐GlcNAc signal in the area of actin (~43 kDa). Similar to total protein lysate, the level of O‐GlcNAcylation was higher in soleus compared to EDL.

### Protein O‐GlcNAcylation in soleus was increased after 6 weeks of treadmill running

**Figure 3:**
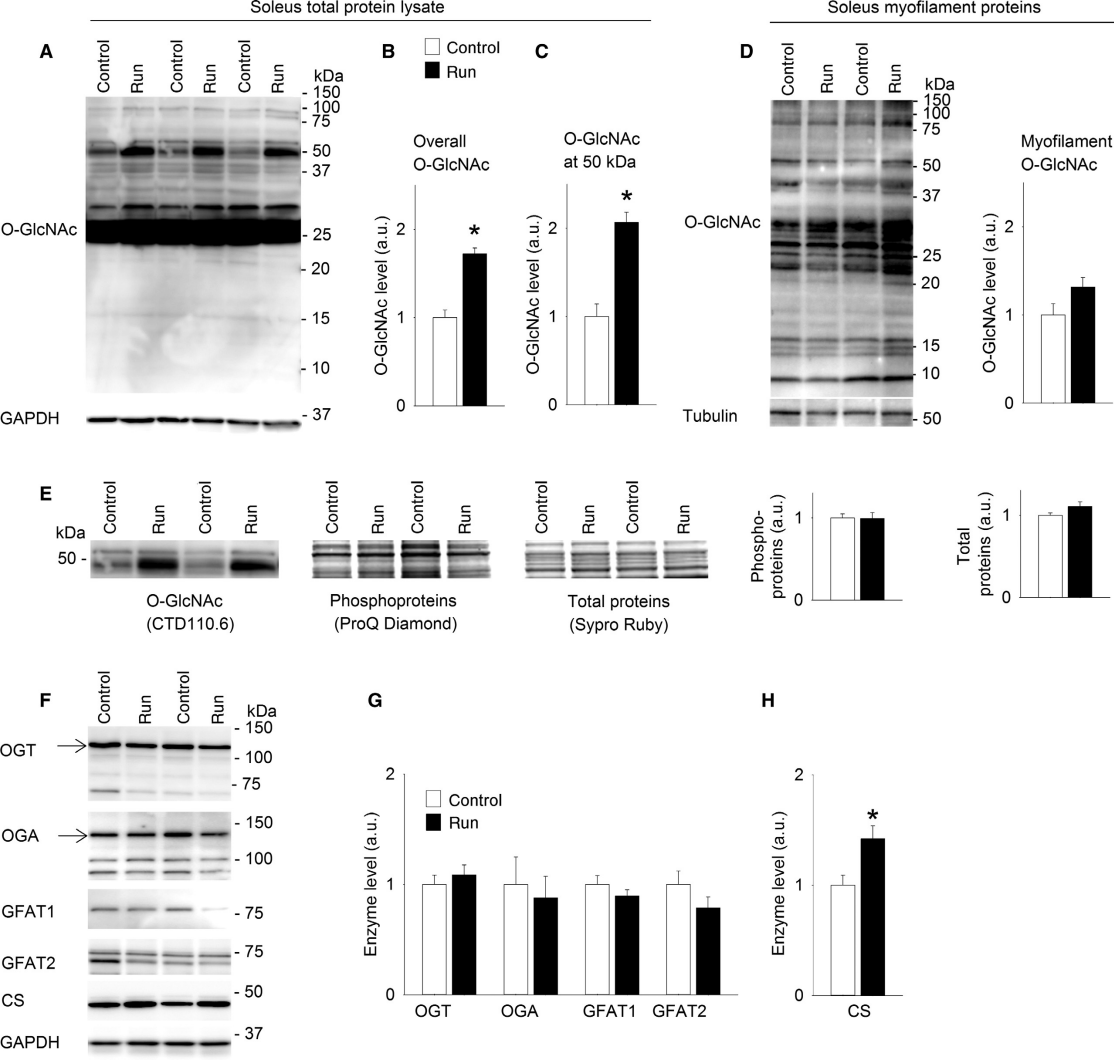
Protein O‐GlcNAcylation in soleus was increased after 6 weeks of treadmill running. Representative immunoblot of O‐GlcNAcylated proteins (A) in total protein lysate from soleus (Sprague Dawley rats) after 6 weeks of exercise (Run) compared to sedate controls (Control). Average data of overall O‐GlcNAcylation (B) and O‐GlcNAcylation at ~50 kDa (C) shown as mean ± SEM, with staining intensities in the exercise group (run, black bars) calculated relative to sedate controls (control, white bars). O‐GlcNAcylation of myofilament proteins did not change after 6 weeks exercise (D), shown as a representative immunoblot and average data. The protein band with increased O‐GlcNAcylation at ~50 kDa in total protein lysate was not accompanied by reciprocal changes in phosphorylation level (ProQ Diamond gel stain) or total protein level (Sypro Ruby gel stain) (E), shown as representative immunoblots/gels and average data. OGT, OGA, GFAT1, GFAT2 and Citrate synthase (CS) are shown as representative immunoblots (F) and average data (G and H). GAPDH level was not altered after exercise training compared to control and was used as loading control for total protein lysates, while tubulin was used for myofilament proteins. Arrows indicate the bands included in the calculation of OGT and OGA (*n = *6. *P < 0.05 vs. sedate controls).

We next investigated whether protein O‐GlcNAcylation in skeletal muscle was regulated by long‐term exercise training, in a rat model of interval training by treadmill running. In total protein lysate from soleus, O‐GlcNAcylation level was increased after 6 weeks of exercise training compared to sedate controls (Fig. 3A and B). Strikingly, a twofold increase in O‐GlcNAcylation was evident at a ~50 kDa protein band after exercise training (Fig. 3A and C). In contrast, investigation of myofilament proteins did not show differences in the O‐GlcNAcylation level in soleus after the training period (Fig. 3D, *P* = 0.10), and further analyses were therefore focused on total protein lysate. The highly O‐GlcNAc‐modified proteins ~50 kDa in total protein homogenate were also investigated for protein phosphorylation and total protein expression (Fig. 3E). There were no reciprocal changes in protein phosphorylation (ProQ Diamond) after 6 weeks exercise. Furthermore, the total protein level (Sypro Ruby) at ~50 kDa was not changed, suggesting increased O‐GlcNAcylation per protein rather than increased amount of the modifiable proteins. Despite the increased protein O‐GlcNAcylation after 6 weeks of exercise, we did not detect altered levels of OGT, OGA, GFAT1, or GFAT2 (Fig. 3F and G). The mitochondrial enzyme citrate synthase (CS) in soleus was increased after the training period (Fig. 3H), reflecting the expected increase in oxidative capacity after long‐term exercise training.

### Protein O‐GlcNAcylation in EDL was increased after 6 weeks of treadmill running

**Figure 4:**
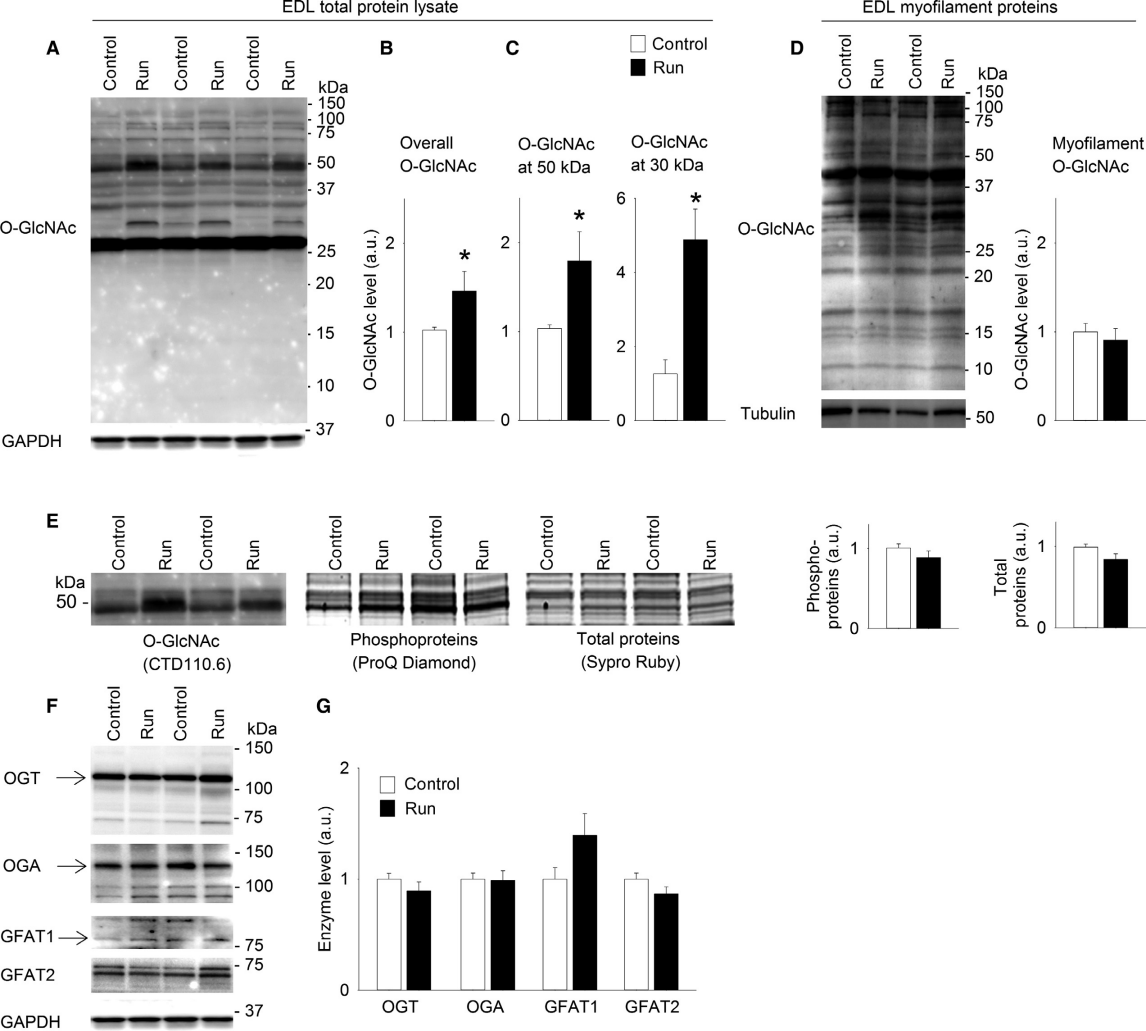
Protein O‐GlcNAcylation in EDL was increased after 6 weeks of treadmill running. Representative immunoblot of O‐GlcNAcylated proteins (A) in total protein lysate from EDL (Sprague Dawley rats) after 6 weeks of exercise (Run) compared to sedate controls (Control). Average data of overall O‐GlcNAcylation (B) and O‐GlcNAcylation at ~50 kDa and at ~30 kDa (C) shown as mean ± SEM, with staining intensities after exercise (Run, black bars) calculated relative to sedate controls (Control, white bars). O‐GlcNAcylation of myofilament proteins in EDL did not change after 6 weeks exercise (D) shown as representative immunoblot and average data. The increased O‐GlcNAcylation at ~50 kDa in total protein lysate was not accompanied by reciprocal changes in phosphorylation level (ProQ Diamond) or total protein level (Sypro Ruby) (E), shown as representative blots/gels and average data. OGT, OGA, GFAT1 and GFAT2 are shown as representative immunoblots (F) and average data (G). GAPDH level was not altered after exercise training compared to control and was used as loading control for total protein lysates, while tubulin was used for myofilament proteins. Arrows indicate the bands included in the calculations of OGT, OGA, GFAT1 (*n =* 6. *P < 0.05 vs. sedate controls).

Similar to soleus, the fast twitch EDL displayed increased O‐GlcNAcylation level after 6 weeks of treadmill running (Fig. 4A and B), analyzed in total protein lysate. There was also increased O‐GlcNAcylation of proteins ~50 kDa in size in EDL after long‐term exercise training (Fig. 4A and C), as well as a manyfold increase in O‐GlcNAcylation of proteins ~30 kDa in size. Investigation of myofilament proteins (Fig. 4D) did not reveal significant changes in the O‐GlcNAcylation level after the training period, and further analyses were focused on total protein lysate. The O‐GlcNAc‐modified proteins at ~50 kDa in total protein lysate were analyzed in parallel for phosphorylation and total protein level (Fig. 4E), but the prominent increase in O‐GlcNAcylation of proteins ~50 kDa in size after 6 weeks exercise was not accompanied by changes in protein phosphorylation pattern or total protein level. Finally, the levels of OGT, OGA, GFAT1 (only weakly expressed), and GFAT2 (Fig. 4F and G) in EDL were not altered after long‐term exercise training.

### Skeletal muscle O‐GlcNAcylation was not altered after one acute exercise bout

**Figure 5:**
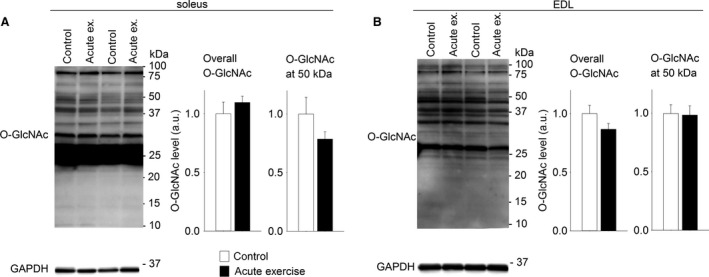
Skeletal muscle O‐GlcNAcylation level was not altered after one acute exercise bout. Representative immunoblots of O‐GlcNAcylated proteins and average data of overall O‐GlcNAcylation and O‐GlcNAcylation at ~50 kDa after one acute exercise bout, analyzed in total protein lysate from soleus (A) and EDL (B) (Wistar rats). Data are mean ± SEM after an acute exercise bout (black bars) relative to sedate controls (white bars). GAPDH level was not altered after acute exercise compared to control and was used as loading control (*n* = 6).

To investigate whether the increase in O‐GlcNAcylation of the proteins ~50 kDa in size could be evoked already after an acute exercise bout, rats performed one bout of exhausting treadmill running. Evidently, overall O‐GlcNAcylation and O‐GlcNAcylation at ~50 kDa were not altered after one acute exercise bout, analyzed in total protein lysate from soleus (Fig. 5A) and EDL (Fig. 5B) harvested immediately after exercise. O‐GlcNAcylation was also analyzed in soleus from rats allowed 24 h of rest after one acute exercise bout (recovery group) before muscle harvesting, and no differences in O‐GlcNAcylation level were detected (recovery group 0.84 ± 0.04 [*n* = 4] vs. sedate controls 1.00 ± 0.07).

### Skeletal muscle O‐GlcNAcylation level was not altered in rats with heart failure

**Figure 6:**
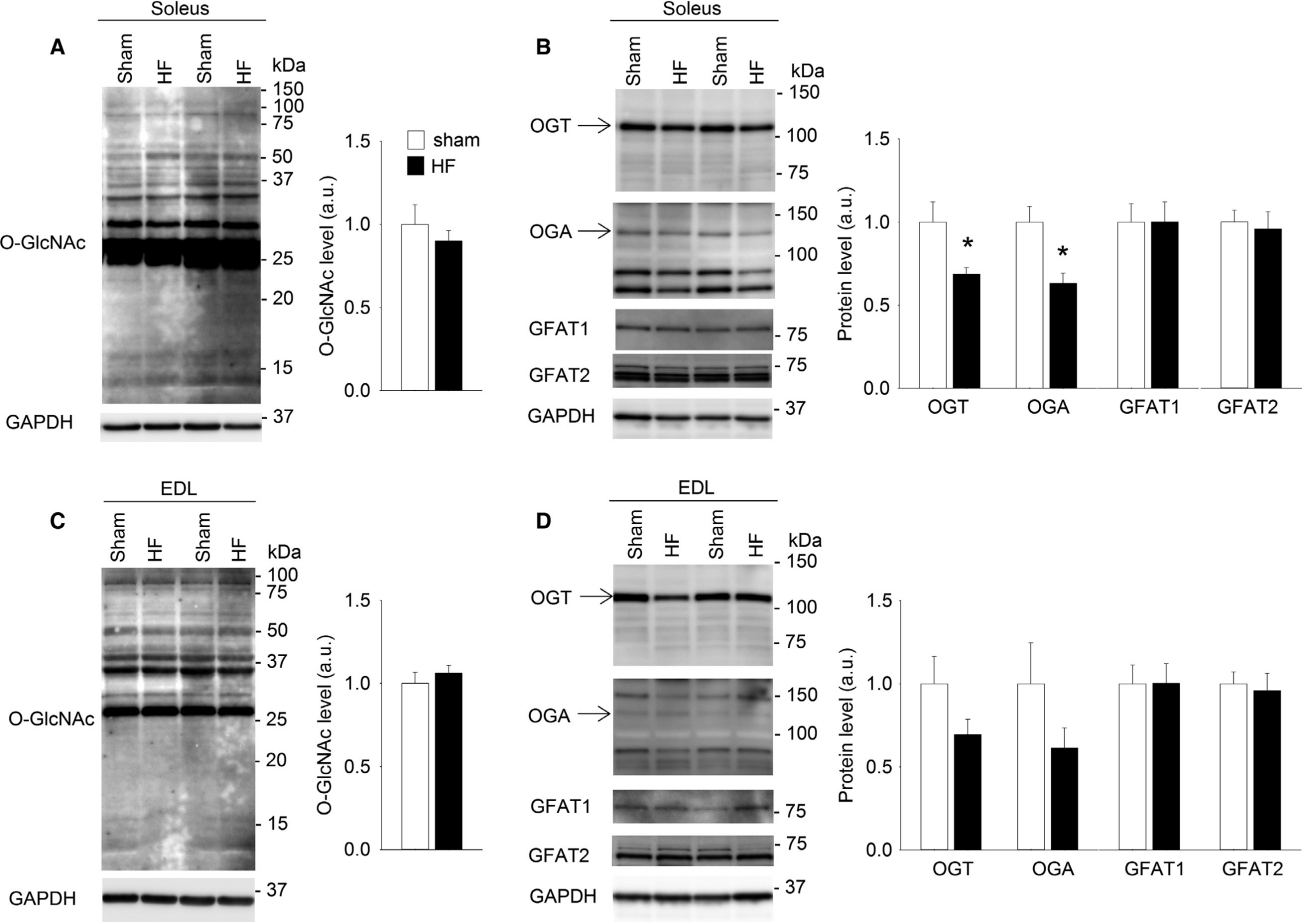
Skeletal muscle O‐GlcNAcylation level was not altered in rats with heart failure. Representative immunoblot of O‐GlcNAcylated proteins and average data of overall O‐GlcNAcylation (A) in total protein lysate of soleus from Wistar rats with postinfarction heart failure (HF, black bars) compared to sham‐operated rats (sham, white bars). Data are shown as mean ± SEM, with staining intensities in HF rats calculated relative to sham rats. OGT, OGA, GFAT1 and GFAT2 in soleus (B) are shown as representative immunoblots and average data. Representative immunoblot of O‐GlcNAcylated proteins and average data of overall O‐GlcNAcylation in EDL total protein lysate (C). OGT, OGA, GFAT1 and GFAT2 in EDL (D) are shown as representative immunoblots and average data. GAPDH level in skeletal muscle was not different between HF and sham‐operated rats and was used as loading control. Arrows indicate the bands included in the calculation of OGT and OGA (*n *=* *7. *P < 0.05 vs. sham).

We next investigated if skeletal muscle protein O‐GlcNAcylation was regulated in postinfarction congestive HF in rats, as a possible explanation for the skeletal muscle dysfunction observed in this pathology. Immunoblotting did not detect significant changes in the O‐GlcNAcylation level in total protein lysate from soleus from HF rats compared to sham‐operated controls (Fig. 6A). However, the levels of the O‐GlcNAc‐modulating enzymes OGT and OGA were reduced in HF compared to sham (Fig. 6B). Also in EDL, protein O‐GlcNAcylation level in HF was not different from sham (Fig. 6C), while the levels of OGT (*P* = 0.14) and OGA (*P* = 0.19) tended to be lower in HF (Fig. 6D). The levels of GFAT1 and GFAT2 were not altered in HF (Fig. 6B and D).

### Human skeletal muscle O‐GlcNAcylation was plentiful and not altered in heart failure

**Figure 7:**
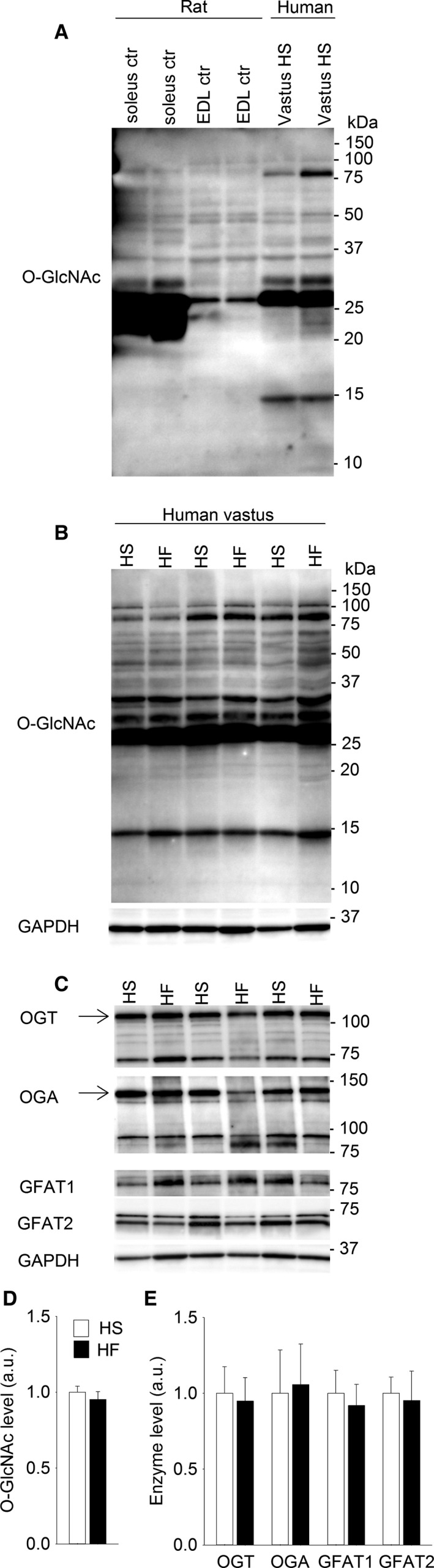
Representative immunoblot of skeletal muscle O‐GlcNAc pattern in rats and humans (A), showing soleus and EDL from Wistar rats compared to human vastus lateralis from healthy subjects (HS) (total protein homogenate). Representative immunoblot of total protein O‐GlcNAcylation in human vastus lateralis from heart failure (HF) patients compared to HS (B). OGT, OGA, GFAT1 and GFAT2 in vastus lateralis from HF patients compared to HS are shown as representative immunoblots (C). Average data from B and C (D, E) shown as mean ± SEM, with staining intensities in HF (black bars) calculated relative to HS (white bars). GAPDH was not different between HF patients and HS and was used as loading control. Arrows indicate the bands included in the calculation of OGT and OGA (*n* = 6. *P < 0.05 vs. HS).

For the analysis of protein O‐GlcNAcylation in human skeletal muscle, we analyzed total protein homogenate from human vastus lateralis (Fig. 7A). There was plentiful O‐GlcNAcylation in human skeletal muscle, and interestingly, the pattern of protein O‐GlcNAcylation in human vastus lateralis (~40% slow type I fibers, ~60% fast type II fibers) (Munkvik et al. 2010) showed similarities with the O‐GlcNAcylation patterns from both soleus (>90% slow type I fibers) and EDL (>90% fast type II fibers) (Soukup et al. 2002) in rat (Fig. 7A).

Subsequently, the O‐GlcNAcylation level in vastus lateralis from healthy subjects (HS) was compared to the O‐GlcNAcylation level in patients with postinfarction HF. The immunoblotting did not reveal differences in overall protein O‐GlcNAcylation in vastus lateralis from HF patients compared to HS (Fig. 7B and D). Furthermore, the O‐GlcNAc‐modulating enzymes OGT, OGA, GFAT1, and GFAT2 were all detected in human vastus lateralis (Fig. 7C), but without differences between HF patients and HS (Fig. 7E).

### Increased O‐GlcNAcylation of cytoplasmic proteins ~50 kDa in size after exercise training

**Figure 8:**
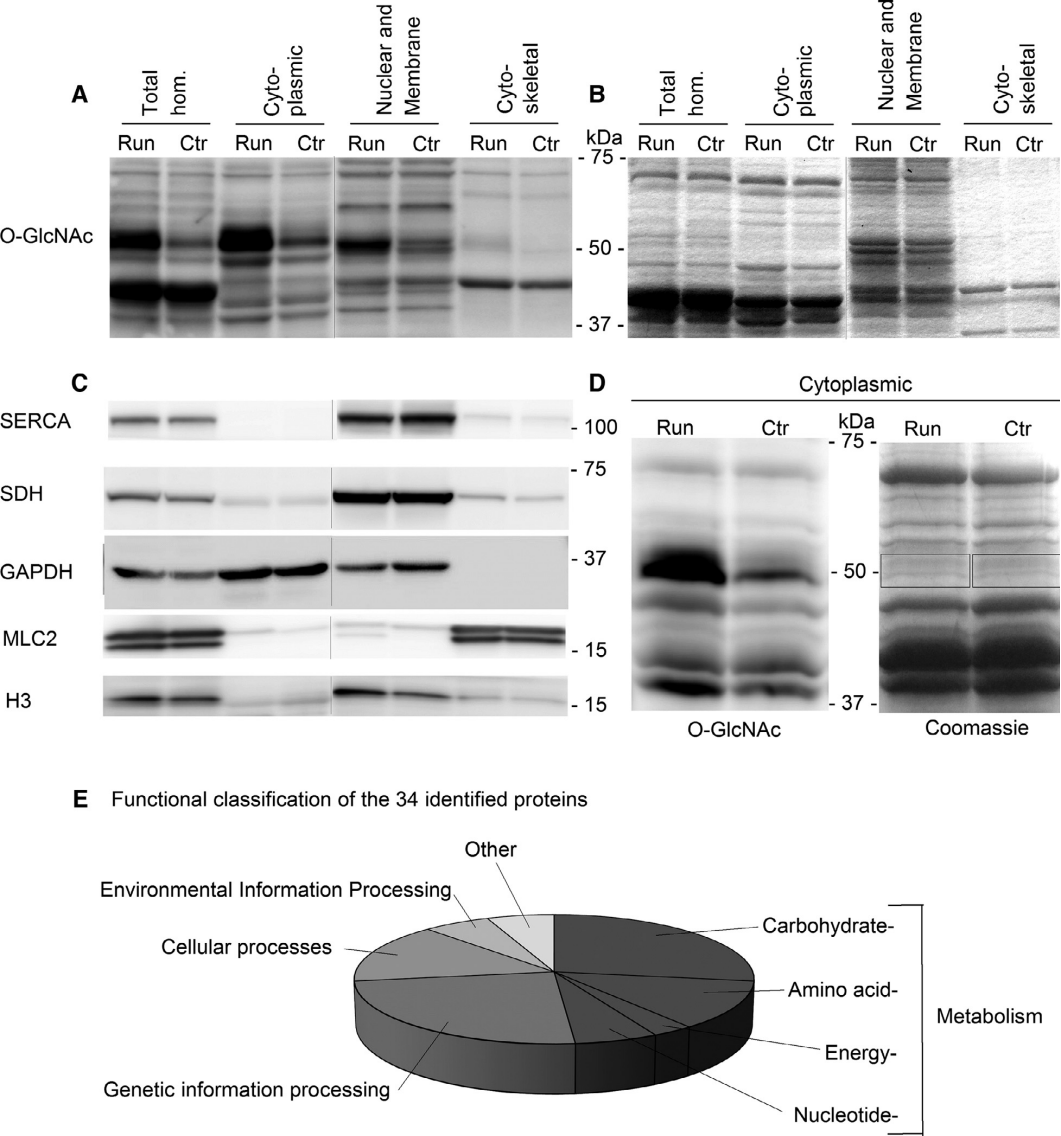
Cytoplasmic proteins ~50 kDa in size with increased O‐GlcNAcylation after long‐term exercise. Immunoblotting with anti‐O‐GlcNAc after subcellular fractioning revealed cytoplasmic localization of the strongly O‐GlcNAc‐modified protein band at ~50 kDa after 6 weeks of exercise (A), analyzed in samples from exercised (run) and control (ctr) soleus (Sprague Dawley rats). A parallel Coomassie‐stained gel (B) verified equal loading of run and ctr. Reprobing of the immunoblot in A with known markers of subcellular compartments confirmed enrichment of the different subcellular fractions (C): SERCA2 for membrane proteins, SDH for mitochondrial proteins, GAPDH for cytoplasmic proteins, MLC2 for myofilament proteins, and H3 for nuclear proteins. Black vertical lines between gel lanes (A–C) mark noncontiguous gel lanes, although within the same gel. The cytoplasmic fraction was analyzed by anti‐O‐GlcNAc in parallel with Coomassie gel staining (D), and the protein bands of interest at ~50 kDa (*boxes*) were cut out for mass spectrometry (MS) analysis. The 34 proteins identified by MS analysis were functionally classified using KEGG BlastKOALA (E), with metabolic proteins (dark gray) representing the largest group.

One main result from the mapping of protein O‐GlcNAcylation in skeletal muscle was the detection of the ~50 kDa protein band with markedly increased O‐GlcNAcylation after 6 weeks of treadmill running. For a more detailed investigation of these O‐GlcNAcylated proteins ~50 kDa in size, we performed enrichment of subcellular protein fractions from soleus muscle from the exercise‐trained rats and sedate controls. The strongly O‐GlcNAc‐modified ~50 kDa protein band in the exercise‐trained rats was mainly detected in the cytoplasmic fraction (Fig. 8A). Equal loading was confirmed by Coomassie staining (Fig. 8B), and enrichment of proteins within the different fractions was confirmed by known markers of subcellular compartments (Fig. 8C). There was some O‐GlcNAc signal at ~50 kDa also in the nuclear and membrane fractions (Fig. 8A) that probably represented overflow of cytoplasmic proteins (cf. Fig. 8C *GAPDH*). Thus, the ~50 kDa proteins with increased O‐GlcNAcylation after long‐term exercise were likely located in the cytoplasm.

Subsequent analysis therefore focused on identifying the O‐GlcNAcylated proteins by cytoplasmic enrichment and SDS‐PAGE separation to reduced complexity. By parallel anti‐O‐GlcNAc immunoblots and Coomassie‐stained gels (Fig. 8D), we obtained precise alignment so that the protein bands of interest could be cut out from the gel for MS analysis. Unfortunately, we did not succeed in detecting GlcNAc reporter ions in the MS2 spectra. However, by the MS analysis and subsequent filtering, we identified 34 candidate proteins present in the strongly O‐GlcNAc modified ~50 kDa band (Table 1). Functional classification (Fig. 8E) of these cytoplasmic proteins revealed that the largest group was metabolic enzymes involved in carbohydrate, amino acid, and nucleotide metabolism, representing possible targets for O‐GlcNAc modifications to link energy availability to energy usage. Interestingly, 10 of the candidate proteins were previously reported to be O‐GlcNAc modified (Table 1), of which creatine kinase, beta‐enolase, and serine/threonine protein phosphatase 2A (55 kDa regulatory subunit B alpha isoform) were detected as O‐GlcNAc modified specifically in rat skeletal muscle (Cieniewski‐Bernard et al. 2004). Several of the other identified enzymes, including pyruvate kinase, were previously detected as O‐GlcNAc modified in HeLa cells (Nandi et al. 2006). Finally, computational prediction of O‐GlcNAc sites using dbOGAP and YinOYang revealed potential O‐GlcNAcylation on all the candidate proteins (Table 1).

**Table 1:** Protein candidates identified by mass spectrometry analysis of the strongly O‐GlcNAc‐modified protein band at ~50 kDa (in rat soleus muscle after 6 weeks of exercise training)

Name	KEGG orthology	Mass Spectrometry data				Published O‐GlcNAc modification	Predicted O‐GlcNAc sites (YinOYang)		Predicted O‐GlcNAc sites (OGlcNAcScan)	
		Molecular weight (kDa)	Peptides	Sequence coverage (%)	PEP	Reference	Serine	Threonine	Serine	Threonine
Beta‐enolase	Carbohydrate metabolism	47	28	62	4.E‐262	Cieniewski‐Bernard et al. (2004)	**37** a, 401	305	115, **37**	395
Alpha‐enolase	Carbohydrate metabolism	47	36	83	0.E+00	Nandi et al. (2006); Park et al. (2007)	**37**, 79, 401		**37**, 63, 115, 176, 249, 427	308, 395, 237
Pyruvate kinase PKM	Carbohydrate metabolism	58	28	58	7.E‐290	Nandi et al. (2006)	**6**, 222, 403	195, **454**, 459	**6**, 55, 67	143, **454**
6‐phosphogluconate dehydrogenase, decarboxylating	Carbohydrate metabolism	53	22	53	4.E‐284	Nandi et al. (2006)	405, **477**, 478, 479	263, **412**,** 424**	**477**, 257	**412**, 50, **424**
Phosphoglycerate kinase 1	Carbohydrate metabolism	45	13	41	2.E‐78	–	**174**, 305, 415	**302**	393, 57, **174**	**302**
Glucose‐6‐phosphate isomerase	Carbohydrate metabolism	63	22	57	0.E + 00	Nandi et al. (2006)	367, 533, 537	168, 536	237	195
UTP–glucose‐1‐phosphate uridylyltransferase	Carbohydrate metabolism	57	24	60	2.E‐206	–	2, 9	398	45	397, 41
4‐Trimethylaminobutyraldehyde dehydrogenase	Carbohydrate metabolism	54	18	43	5.E‐106	–	2, 9, 188, **229**	3, 5	**229**, 46	312
Phosphoglucomutase 1	Carbohydrate metabolism	61	24	46	2.E‐144	Nandi et al. (2006)	247, **338**,** 541**	19, 96, 144, 326, 556, 559, 562	547, **338**, 309, 20, 151, **541**, 168, 505, 191	9
S‐adenosylmethionine synthase isoform type‐2	Amino acid metabolism	44	10	27	1.E‐52	–	**293**,** 319**,** 325**	182, **185**,** 374**	**319**, 338, **293**,** 325**	**374**,** 185**
Aspartate aminotransferase, cytoplasmic	Amino acid metabolism	46	14	42	1.E‐136	–	5, 46, 137, 297	132, 410	404	403
Cytosolic nonspecific dipeptidase	Amino acid metabolism	53	16	39	5.E‐112	–	354, 358, 387, 439, 471	270	358, 324, 87, 341, 130, 32	150
Creatine kinase M‐type	Amino acid metabolism	43	21	53	2.E‐185	Cieniewski‐Bernard et al. (2004)	122, 199			327
Alanine aminotransferase 1	Energy metabolism	55	18	48	4.E‐243	–	3, 9, **301**, 363, 368, 496	302, 492	**301**, 124, 173	
Adenylosuccinate lyase	Nucleotide metabolism	55	19	54	1.E‐196	–	4, 359		474, 257, 407	239
Adenylosuccinate synthetase isozyme 1	Nucleotide metabolism	50	17	49	2.E‐165	–	2, 31, 61, 173, 272, 428	**14**, 271, 332, 414	87	**14**
Hsc70‐interacting protein	Genetic information processing	41	10	26	6.E‐67	–	7, 273		51	
Protein Smyd1	Genetic information processing	57	10	28	4.E‐37	–	**162**, 358	426	243, **162**	
Protein Smyd5	Genetic information processing	47	3	8	4.E‐17	–	**29**, 140	**117**,** 258**, 415	**29**, 22, 260	176, **117**, 164, **258**, 232, 251
Protein Ruvbl2	Genetic information processing	51	14	37	9.E‐72	–	463	8, 81, **152**, 154, 338, **359**, 459, 462, 463	262, 203	120, **152**,** 359**
NEDD8‐activating enzyme E1 catalytic subunit	Genetic information processing	52	17	49	3.E‐187	–	194, 315	60, 316, 419, **450**, 451		**450**, 302, 403, 353
Spliceosome RNA helicase Ddx39b	Genetic information processing	49	13	29	5.E‐54	–	40, 145, 426	**111**, 427	38, 421, 41, 130	**111**
Elongation factor 1‐alpha 1‐like	Genetic information processing	50	12	33	1.E‐61	Nandi et al. (2006)	**107**, 163	187, **217**, 279, 286, 287, **452**	194, 300, 396, **107**, 175	**452**, 432, **217**
COP9 signalosome complex subunit 2	Genetic information processing	52	23	58	2.E‐158	–	440	104	268	356, 135
cAMP‐dependent protein kinase type II‐alpha reg. subunit	Cellular processes	46	10	35	3.E‐40	–	48, 63, 64,	56, 57, 93, **186**, 221, 340	97	**186**
Myc box‐dependent‐interacting protein 1	Cellular processes	65	13	36	2.E‐111	–	304, 321, 386, 426, 478, **482**	328, **475**, 584	288, 16, **482**, 176, 332	23, 492, **475**
Annexin A7	Cellular processes	50	9	21	8.E‐54	–	24, 25, 29, 51, 109, 137, 450	9, 420, **440**, 443	291	**440**, 434
Rab GDP dissociation inhibitor beta	Cellular processes	51	10	29	3.E‐73	Nandi et al. (2006)	65	**122**,** 166**	354, 242, 338	407, 355, **122**,** 166**, 94
Alpha‐1‐antiproteinase	Cellular processes	46	16	43	2.E‐110	–	4, 33, 38	32	310, 6, 231, 54	252
Ser/thr‐protein phosphatase 2A 55 kDa reg. subunit B delta	Environmental information processing	52	8	21	3.E‐37	–	282, 288, 293,	384	292, 119, 131, 300	308
Ser/thr‐protein phosphatase 2A 55 kDa reg. subunit B alpha	Environmental information processing	52	19	46	2.E‐145	Cieniewski‐Bernard et al. (2004)	75	148, 150, 436	286, 125, 294, 113	302
Serine protease inhibitor A3N	Other	46	12	37	7.E‐151	–	**76**, 192, 217, **290**	155, **257**, 283, 358, 364	305, 161, **290**,** 76**, 67	**257**, 191
Aspartyl aminopeptidase	Other	53	15	44	2.E‐93	–	**147**, 279, 340, 383		**147**, 340, 231, 387, 344, 324	
Tetratricopeptide repeat protein 38	Not found	52	21	60	1.E‐163	–	160, **175**, 176, 427	169, 172, 208, 426	144, 183, 96, 243, **175**	459, 75

a Bold letters indicate sites identified by both YinOYang and OGlcNAcScan.

## Discussion

In this study, we showed extensive protein O‐GlcNAcylation in skeletal muscle that was dynamically regulated by exercise training. The O‐GlcNAcylation level was higher in muscles with predominantly type I fibers compared to type II fibers, and this fiber type difference was related to the expression of OGT, OGA, and GFAT. Six weeks of exercise training markedly increased O‐GlcNAcylation on a subgroup of cytoplasmic proteins, suggesting that O‐GlcNAc signaling on cytoplasmic proteins is part of the training response. In contrast, postinfarction HF did not affect the O‐GlcNAcylation level in skeletal muscle, indicating that aberrant O‐GlcNAcylation cannot explain the skeletal muscle dysfunction observed in HF. From a translational point of view, an important finding was that comparable O‐GlcNAcylation patterns appeared in rat and human skeletal muscle.

### High O‐GlcNAcylation level in slow twitch soleus muscle

Strikingly, overall O‐GlcNAcylation level detected by immunoblotting was higher in the prototypical slow twitch soleus muscle compared to fast twitch EDL, consistent with previous measurements by radioactive labeling (Cieniewski‐Bernard et al. 2004, 2006). The finding of higher levels of OGT, GFAT1, and GFAT2 (all in favor of increased O‐GlcNAcylation) in soleus compared to EDL indicates a fiber–type‐specific (type I vs. type II fibers) expression of the O‐GlcNAc‐modulating enzymes. It has been estimated from adipocytes that 2–5% of incoming glucose is diverted to the HBP (Marshall et al. 1991). However, the observed differences in enzyme levels of GFAT between slow and fast twitch skeletal muscles suggest that the estimate may not hold true in all tissues. GFAT is the rate‐limiting enzyme in the HBP, and remarkably both isoforms GFAT1 and GFAT2 were three times higher in soleus compared to EDL. This suggests that more of the available glucose can enter the HBP in soleus, consistent with our finding of higher O‐GlcNAcylation level in this muscle compared to EDL. To our knowledge, we are the first to quantify GFAT1 and GFAT2 protein levels in skeletal muscle, and the detection of both isoforms is supported by previous mRNA analysis (Oki et al. 1999). However, the enzyme activities should also be clarified. Interestingly, the level of OGA (removing the O‐GlcNAc from proteins) was also higher in soleus compared to EDL, likely a required counterpart in the dynamic modulation of high O‐GlcNAcylation levels.

Many O‐GlcNAcylated proteins are also phosphoproteins, and the two PTMs have been shown to interplay by both competitive and noncompetitive regulation (Wang et al. 2008; Hart et al. 2011). We found that in skeletal muscle, both the O‐GlcNAcylation and the serine and threonine phosphorylation signals were most prominent on proteins 20–50 kDa in size. The high level of both PTMs in this protein range might imply interaction between phosphorylation and O‐GlcNAcylation, and this remains to be investigated on individual proteins. Since our analyses were optimized for proteins 20–100 kDa in size, modifications by O‐GlcNAcylation or phosphorylation on proteins >100 kDa could have escaped detection.

### Exercise training increases protein O‐GlcNAcylation in skeletal muscle

This is the first study to investigate the effects of long‐term exercise training on O‐GlcNAcylation in skeletal muscle. Our results suggest that O‐GlcNAc signaling participates in the training response by increasing protein O‐GlcNAcylation, in both slow and fast twitch muscles. Interestingly, inactivity that caused muscle atrophy, as the counterpart to exercise, was associated with decreased O‐GlcNAcylation in rat soleus muscle (Cieniewski‐Bernard et al. 2006). Reduction in O‐GlcNAcylation was also found after 60 days of bed rest in human vastus lateralis (Mounier et al. 2009), and the reduction was prevented by light exercise. Together with the results from the present study, these findings suggest a dynamic modulation of protein O‐GlcNAcylation in response to the activity level of the muscle.

Furthermore, the increased O‐GlcNAcylation after 6 weeks exercise training seems to be a result of repeated exercise, because an acute exercise bout did not alter the O‐GlcNAcylation level in soleus or EDL. Consistent with this finding, a recent study (Peternelj et al. 2015) reported unaltered O‐GlcNAcylation in soleus muscle after acute exercise by treadmill running. EDL was not investigated in that study, but white gastrocnemius muscle (mainly fast twitch fibers) showed increased O‐GlcNAcylation after acute exercise, in some contrast to our findings of unaltered O‐GlcNAcylation in EDL. The authors suggest that the increased O‐GlcNAcylation in gastrocnemius was due to increased oxidative stress (Peternelj et al. 2015), and this should be clarified in further studies. In cardiac muscle, both reduced (Belke 2011) and increased (Cox and Marsh 2013) O‐GlcNAc levels have been reported in different exercise models. Hence, there is currently no consensus on the effect of exercise on O‐GlcNAcylation across different muscle types and exercise modes. The present data strongly suggest that O‐GlcNAcylation is increased after long‐term interval training, in both fast and slow twitch skeletal muscles.

Despite the increased protein O‐GlcNAcylation after 6 weeks of exercise training, the level of the O‐GlcNAc modulating enzymes OGT, OGA, GFAT1, and GFAT2 did not change. This is in accordance with several other studies that have reported altered protein O‐GlcNAcylation without concomitant changes in total enzyme levels (Cieniewski‐Bernard et al. 2006; Medford et al. 2013; Peternelj et al. 2015; Ramirez‐Correa et al. 2015). Importantly, we report increased O‐GlcNAcylation of a *subgroup* of cytoplasmic proteins after exercise training, suggesting highly targeted regulation of OGT and OGA toward their substrates. The regulation and targeting of OGT and OGA is complex and not fully understood (Nagel and Ball 2014), and dedicated studies are warranted to better understand the upstream regulation and targeting of OGT and OGA in exercise.

We suggest that increased substrate flux could be a trigger in the series of events leading to the increased O‐GlcNAcylation observed after 6 weeks of exercise training. Exercise training increases expression of insulin‐ and contraction‐regulated glucose transporter isoform 4 (GLUT4), resulting in improved insulin sensitivity and increased glucose uptake in the trained muscle (Richter and Hargreaves 2013). The increased glucose uptake is likely to increase the flux through the HBP, resulting in increased UDP‐GlcNAc, supported by the increase in UDP‐GlcNAc previously reported after acute exercise (Nelson et al. 1997). Increased UDP‐GlcNAc enhances the activity of OGT (Kreppel and Hart 1999), and hence the increased glucose uptake could be the initiator of increased protein O‐GlcNAcylation after exercise training. Interestingly, contrary to exercise training that has many beneficial effects, pathological nutrient excess and hyperglycemia have also been associated to excessive O‐GlcNAcylation in skeletal muscle (Yki‐Jarvinen et al. 1998; Arias et al. 2004). Yang et al. (2008) linked increased O‐GlcNAcylation of proteins in the insulin signaling pathway to compromised insulin sensitivity. We speculate that in exercise, dynamic O‐GlcNAcylation may provide optimal kinetics of metabolic pathways. On the contrary, energy excess in the sedentary could cause overshoot of O‐GlcNAc signaling which seems detrimental.

In the myofilament protein fraction, the different O‐GlcNAcylation pattern in soleus compared to EDL supports a modulatory role of O‐GlcNAc in the contractile apparatus, as recently suggested in both skeletal (Hedou et al. 2007; Cieniewski‐Bernard et al. 2012, 2014; Stevens et al. 2013; Hortemo et al. 2015) and cardiac (Ramirez‐Correa et al. 2008, 2015) muscle. Furthermore, our research group (Hortemo et al. 2015) and Cieniewski‐Bernard et al. (2014) recently showed that OGT and OGA are associated with myofilament proteins. We did not detect alterations in O‐GlcNAcylation level of myofilament proteins after treadmill running, although there was a trend toward increased O‐GlcNAcylation in soleus after 6 weeks of treadmill running. Methodologically, the O‐GlcNAc antibody might not be sensitive enough to detect subtle changes in O‐GlcNAcylation of myofilament proteins by western blotting, and more targeted analyses seems necessary (Cieniewski‐Bernard et al. 2014; Hortemo et al. 2015; Ramirez‐Correa et al. 2015) to detect O‐GlcNAc dynamics on myofilament proteins in future studies.

### Skeletal muscle protein O‐GlcNAcylation in heart failure in rats and humans

Postinfarction congestive HF is related to reduced exercise tolerance and skeletal muscle dysfunction (Zizola and Schulze 2013). In the present study, the investigation of skeletal muscle O‐GlcNAcylation did not reveal differences between HF and healthy controls, neither in rats nor in humans. This indicates that modification of skeletal muscle O‐GlcNAcylation level cannot explain the reduced exercise tolerance in HF. However, the reduced levels of OGA and OGT in soleus (and the same tendency in EDL) from HF rats could suggest downregulated O‐GlcNAc cycling of selected proteins, despite overall levels being unchanged.

To our knowledge, we are the first to investigate skeletal muscle O‐GlcNAcylation in HF. In cardiac muscle, increased protein O‐GlcNAcylation has been reported in cardiac pressure overload and HF (Lunde et al. 2012), and increased protein O‐GlcNAcylation is involved in development of diabetic cardiomyopathy (reviewed by Marsh et al. 2014). In these cardiac pathologies, increased glucose availability is suggested as a key mediator to the increased O‐GlcNAcylation. There is controversy regarding the extent of metabolic adaptations in skeletal muscle in HF (Rehn et al. 2012; Zizola and Schulze 2013), and the etiology of HF is likely to play a role. Despite the evident differences between the rats with HF and the human HF patients in the present study (e.g., young animals vs. old humans, MI is induced acutely versus following gradually developing cardiac disease), both models represent the same type of HF, that is, postinfarction HF with reduced ejection fraction (HFrEF). Hence, our results indicate that aberrant skeletal muscle O‐GlcNAcylation is not a significant contributor to the pathophysiology of HFrEF.

Importantly, the analysis of human vastus lateralis showed that there is plentiful protein O‐GlcNAcylation in human skeletal muscle. Vastus lateralis is a mixed muscle with regard to fiber type (~40% type I and ~60% type II fibers) (Munkvik et al. 2010), and protein O‐GlcNAc patterns from both soleus (>90% type I fibers) and EDL (>90% type II fibers) from rat (Soukup et al. 2002) were recognized in vastus lateralis. Hence, it seems that skeletal muscle O‐GlcNAcylation is related to the fiber type composition, both in rats and humans. Furthermore, the O‐GlcNAc modulating enzymes were detected by the same antibodies in human and rat skeletal muscle. These results support highly conserved O‐GlcNAc signaling in humans, providing an exciting basis for further studies.

### Candidate proteins of the strongly O‐GlcNAc modified protein band at ~50 kDa

An interesting finding in the analysis of skeletal muscle protein O‐GlcNAcylation was the proteins ~50 kDa in size with markedly increased O‐GlcNAcylation after 6 weeks of treadmill running, both in soleus and EDL. There was also a manyfold increase in O‐GlcNAcylation of proteins ~30 kDa in size, evident only in EDL, that remains to be investigated. We focused on the O‐GlcNAc‐modified proteins ~50 kDa in size, present in both muscle types.

The modified proteins ~50 kDa in size were located in cytoplasm, and we hypothesize that they could represent one or more key metabolic enzymes. Intriguingly, increased O‐GlcNAcylation of proteins ~50 kDa in size has been reported also in pressure overloaded cardiac muscle (Lunde et al. 2012). It is tempting to speculate that it could be the same key metabolic enzymes that are dynamically O‐GlcNAc modified when the muscle is adapting to new demands in exercise or disease, coupling energy availability to energy usage. In support of this hypothesis, the MS analysis of the O‐GlcNAc‐modified protein band at ~50 kDa identified several metabolic enzymes with known (Cieniewski‐Bernard et al. 2004; Nandi et al. 2006; Park et al. 2007) or theoretical O‐GlcNAc modifications. Identification of O‐GlcNAcylated proteins is challenging (Ma and Hart 2014), and unfortunately we did not succeed to confirm the O‐GlcNAc moiety by the MS analysis. However, three of the candidate proteins identified by the MS analysis (creatine kinase, serine/threonine protein phosphatase 2A (55 kDa regulatory subunit B alpha isoform) and beta‐enolase) were previously reported to be O‐GlcNAc modified specifically in rat skeletal muscle (Cieniewski‐Bernard et al. 2004), and are indeed interesting candidates. Furthermore, the computational prediction of O‐GlcNAc sites by dbOGAP and YinOYang revealed potential O‐GlcNAcylation on all the protein candidates identified by MS. There was, however, considerable discrepancy between the predicted O‐GlcNAc sites generated from dbOGAP compared to YinOYang, implying that sites generated from presently available computational predictions need to be validated experimentally. In summary, the proteins ~50 kDa in size with increased O‐GlcNAcylation could represent one or more key metabolic enzymes that are dynamically O‐GlcNAc modified after long‐term exercise training. However, since we could not confirm the O‐GlcNAc moiety by MS analysis, the enzyme identity and the specific O‐GlcNAc sites remains to be clarified.

## Conclusion

In conclusion, we showed extensive and fiber‐type‐related protein O‐GlcNAcylation in skeletal muscle in rats and humans. Six weeks of exercise in a rat model of interval training increased protein O‐GlcNAcylation in skeletal muscle, with markedly increased O‐GlcNAcylation on a subgroup of cytoplasmic proteins. This suggests that dynamic O‐GlcNAc signaling on cytoplasmic proteins is part of the training response. In contrast, postinfarction heart failure did not affect O‐GlcNAcylation level in rat or human skeletal muscle, indicating that aberrant O‐GlcNAcylation cannot explain the skeletal muscle dysfunction in heart failure. Our findings provide novel insight into O‐GlcNAc signaling in skeletal muscle, and further studies are warranted to investigate the functional consequences of skeletal muscle O‐GlcNAcylation in health and disease.

## Conflict of Interest

None declared.
